# Determination of the energy contents and nutrient digestibility of corn, waxy corn and steam-flaked corn fed to growing pigs

**DOI:** 10.5713/ajas.18.0713

**Published:** 2019-02-14

**Authors:** Dongli Ma, Juntao Li, Chengfei Huang, Fengjuan Yang, Yi Wu, Ling Liu, Wei Jiang, Zhicheng Jia, Peijun Zhang, Xuezhen Liu, Shuai Zhang

**Affiliations:** 1 State Key Laboratory of Animal Nutrition, Ministry of Agriculture Feed Industry Centre, College of Animal Science and Technology, China Agricultural University, Beijing 100193, China; 2 College of Agronomy and Biotechnology, China Agricultural University, Beijing 100193, China; 3 Baotou Beichen Feed Sci-tech Co., Ltd. Baotou, Inner Mongolia Autonomous Region 014040, China

**Keywords:** Available Energy, Nutrient Digestibility, Growing Pig, Steam-flaked Corn, Waxy Corn

## Abstract

**Objective:**

The research was conducted to determine the digestible (DE) and metabolizable energy (ME) contents as well as the apparent total tract digestibility (ATTD) of nutrients in corn, waxy corn and steam-flaked corn fed to growing pigs.

**Methods:**

Eighteen growing pigs with initial body weight of 15.42±1.41 kg were randomly allotted to three diets including a corn diet, a waxy corn diet and a steam-flaked corn diet in a completely randomized design. Each treatment contained six replicates. The experiment lasted for 12 days, which comprised 7-d adaptation to diets followed by a 5-d total collection of feces and urine. The energy contents and the nutrient digestibility in three ingredients were calculated using direct method.

**Results:**

Compared to normal corn, both the amylose and dietary fiber contents in waxy corn were numerically lower, but the starch gelatinization degree was numerically greater. Moreover, the DE and ME contents as well as the ATTD of neutral detergent fiber and acid detergent fiber (ADF) in waxy corn were significantly greater (p<0.05) than those in normal corn when fed to growing pigs. Furthermore, the steam-flaked corn had greater (p<0.05) DE and ME contents, and ATTD of ether extract and ADF compared to normal corn.

**Conclusion:**

Both variety and processing procedure have influence on chemical compositions, energy contents and nutrient digestibility of corn. The waxy corn and steam-flaked corn had greater degree of starch gelatinization and DE and ME contents compared to normal corn when fed to growing pigs.

## INTRODUCTION

Corn is widely used in animal feeds due to its high carbohydrate content, good palatability and great energy value. Therefore, a small fluctuation in corn’s energy value greatly affects the energy content of the whole diet. Precision evaluation of the energy and nutrient value of corn and improving the utilization efficiency of corn produces great economic benefit for the swine and poultry industries.

There are many factors that can affect the nutritional value of corn including varieties, habitats and processing procedures [1,2]. Many studies have focused on the nutrient values of different corn varieties such as high-oil [3,4], high lysine [5], or low-phytate [6] corn. Li et al [1] reported that variety had a significant impact on the nutritive values of corn in growing pigs, and they suggested that the variety of corn should be considered when fed to growing pigs. Different corn varieties have different cell wall structure, endosperm type and crystal structure of starch, all of which could affect the nutrient digestion and absorption of the corn. Waxy corn is the only corn variety that originated from China which has large yield especially in northeast China [7]. Waxy corn has good palatability, thus traditionally it has been used as human fresh food and canned food. Now it is gradually being utilized in feed industry, but its nutrient values for growing pigs remains unknown.

Processing procedure is another factor that can affect the nutritional value of corn. With processing, the protein matrix in corn grain and the endosperm type in corn could be partially or completely changed [8]. The steam flaking treatment can increase the starch digestion compared with the dry rolling treatment in cattle [9]. Improved starch digestibility in corn after steam flaking treatment is due to physical damage of the starch-protein matrix and granule structure and starch gelatinization [10]. As a result, steam-flaked corn is widely used as a feed for ruminants [9]. However, few studies have focused on the digestible (DE) and metabolizable energy (ME) values and nutrient digestibility of the steam-flaked corn fed to growing pigs.

Therefore, the objective of this study was to evaluate the DE and ME contents as well as the nutrient digestibility of the waxy corn and steam-flaked corn when fed to growing pigs.

## MATERIALS AND METHODS

The protocol used in this experiment was approved by the Institutional Animal Care and Use Committee of China Agricultural University (Beijing, China). The animal trial was conducted in the Animal Test Base of the National Feed Engineering Technology Research Center (Beijing, China).

### Ingredients

Normal corn and steam-flaked corn used in this experiment were provided by Baotou Beichen Feed Sci-tech Co., Ltd (Baotou, China). Waxy corn (Xiandanuo 001) was provided by Beijing Youzhongyouzai Technology Service Co., Ltd (Beijing, China). The steam-flaked corn was produced using the same batch as the normal corn. The production process is as follows: after cleaning, the corn was fed into a vertical stainless steel steam box and treated with steam at 100°C to 110°C for 30 to 60 minutes. When the moisture content of the maize reached 21% to 23%, it was rolled into thin sheets with large pre-heated two-way stripe rollers (the distance between the two rollers can be adjusted according to the actual need), and then dried to prevent mildew. The production was stored in a dry and ventilated place.

### Animal, diets, and experimental design

Eighteen crossbred barrows (Duroc×Landrace×Yorkshire) with initial body weight of 15.42±1.41 kg were randomly assigned into three treatment diets in a completely randomized design with six pigs per dietary treatment. The treatment diets were formulated with 96.3% normal corn, waxy corn or steam-flaked corn and 3.7% vitamins and minerals (Table 1). The diets were formulated to meet or exceed the nutrient requirements of growing pigs suggested by Nutrient Requirements of Swine (2012). All pigs were individually housed in stainless-steel metabolism crates (1.4 m×0.5 m×0.6 m) and the room temperature was controlled at 22°C±2°C. One week was allowed for all pigs to adapt to the crates before allotted to three treatments, and commercial corn-soybean meal diet was used during this period. The experiment lasted for 12 days which included a 7-d adaptation period for treatment diets followed by a 5-d total collection of feces and urine. During the whole experiment, pigs had free access to water through a low-pressure nipple drinker, and the daily amount of feed provided equaled to 4% of their BW, which were divided into two equal meals and supplied at 0800 and 1600 h [11]. The amount of feed provided and leftovers were recorded at each meal to calculate daily feed consumption.

### Sample collection

All feces defecated during the 5-d collection period were collected into plastic bags as soon as they appeared in the metabolism crates and were immediately stored in −20°C. The collection started at 0800 on d 8 and ended at 0800 on d 13. At the end of the experiment, the 5-d fecal collection from each pig was thawed, mixed and weighed. Approximately 350 g of subsample was taken and dried in a forced-air drying oven at 65°C for 72 h [12]. After drying, subsamples were weighted and the moisture proportion in feces was calculated. Then the total weight of dry feces was estimated based on determined moisture proportion. Subsamples were ground through a 0.25 mm screen (60 mesh) and then kept for further chemical analysis.

All urine excreted by each pig during the 5-d collection period was collected into plastic buckets through a funnel installed under the metabolism crates. Approximately 50 mL of 6 *N* HCl was added to the buckets to limit microbial growth and to reduce the loss of ammonia [13]. The volume of the urine excreted from each pig was recorded every day and a subsample of 10% of the fully mixed urine was transferred into a screw-capped bottle and then stored at −20°C. At the end of the experiment, all kept urine samples were mixed and filtered into 50 mL centrifuge tubes, then stored in −20°C for further analysis.

### Chemical analysis

All three corn samples used in this study were analyzed for bulk density [14], dry matter (DM; Method 934.01) [15], crude protein (CP; Method 990.03) [15], ether extract (EE), crude ash (Method 942.15) [15], calcium, phosphorus (Method 985.01) [15], and starch (Method 948.02) [15]. In addition, neutral detergent fiber (NDF) and acid detergent fiber (ADF) were determined following a modified procedure of van Soest et al [16]. The concentration of NDF was analyzed using heat stable α-amylase and sodium sulfite without correction for insoluble ash as adapted for an Ankom Fiber Analyzer (Ankom Technology, Macedon, NY, USA). The ADF fraction was analyzed in a separate sample. Total dietary fiber (TDF), soluble dietary fiber (SDF) and insoluble dietary fiber (IDF) were determined by the Method 993.43 [17]. Total, insoluble and soluble non-starch polysaccharides (NSP) and their constituent sugars were determined by alditol acetates using a gas-liquid chromatography (GLC) for neutral sugars and a colorimetric method for uronic acids with a modification of the Uppsala method [18] according to Bach Knudsen [19]. The concentration of constituent sugars analyzed by GLC was conducted by an Agilent GC 6890 (Agilent Technologies Co. Ltd., Santa Clara, CA, USA) with a flow of 20 mL/min and split 40:1. A 30 m×0.25 mm×0.25 mm column (Agilent DB-225, film thickness 0.25 μm, Agilent Technologies Co. Ltd., USA) was used at 220°C for column temperature and at 250°C for the injector and detector temperature. The gross energy (GE) of three ingredients were analyzed using an isoperibol oxygen bomb calorimeter (Parr Instruments Co., Moline, IL, USA). The determination of starch gelatinization degree refers to the simplified method of enzymatic hydrolysis suggested by Xiong et al [20]. In this method, the starch gelatinization degree is expressed as the ratio of the amount of glucose released from processed samples to the amount of glucose released from fully ripened samples from the same source. Diets and fecal samples were analyzed for DM, CP, EE, crude fiber, NDF, ADF, ash, calcium, phosphorus, starch and GE. Urine samples were analyzed for CP and GE.

### Calculations

The DE and ME contents of the three ingredients were calculated according to the direct method [11] followed the equations below:

$$\begin{array}{l} \text{The DE content in diet }(\text{kcal}/\text{kg})=(\text{GE in feed intake }[\text{kcal}]-\text{GE in feces }[\text{kcal}])/\text{feed intake }(\text{kg})\\ \text{The ME content in diet }(\text{kcal}/\text{kg})=(\text{GE in feed intake }[\text{kcal}]-\text{GE in feces }[\text{kcal}]-\text{GE in urine }[\text{kcal}])/\text{feed intake }(\text{kg})\\ \text{The DE or ME content in corn }(\text{kcal}/\text{kg})=\text{DE or ME in diet }(\text{kcal})/\text{kg}/0.963 \end{array}$$

### Statistical analysis

All data were checked for normality using UNIVERIATE procedure of SAS 9.1 (SAS Inst. Inc., Cary, NC, USA), and then were analyzed with one-way analysis of variance using general linear model procedure of SAS. An individual pig was treated as the experimental unit and the dietary treatment was the only fixed effect in the model. Treatment means were calculated using LSMEANS statement of SAS, and then were separated using Student-Newman-Keuls test as an adjustment. Statistical significance was declared as p<0.05, and a significant tendency was declared as 0.05≤p<0.1.

## RESULTS

Compared to the normal corn, the amylose content was numerically lower in waxy corn, and the EE content was slightly greater in waxy corn (Table 2). The contents of other analyzed nutrients were close between these two corns. After steam flaking, the bulk density of the normal corn decreased and the content of EE decreased. The contents of other analyzed components were close for normal corn before and after steam flaking.

Compared to the normal corn, the contents of IDF, SDF, and TDF were numerically lower in waxy corn, but the starch gelatinization degree was twice greater in waxy corn (Table 3). After steam flaking, the contents of soluble NSP in general increased in steam-flaked corn, such as soluble arabinose, xylose and galactose, but the content of soluble mannose showed an opposite situation numerically. The steam flaking procedure increased the starch gelatinization degree in normal corn.

The DE and ME contents on DM basis were greater (p<0.05) in waxy corn diet and steam-flaked corn diet than those in normal corn diet fed to growing pigs. The DE and ME contents were greater (p<0.05) in waxy corn and steam-flaked corn compared to those in normal corn in growing pigs (Table 4). For the nutrient digestibility, the apparent total tract digestibility (ATTD) of NDF and ADF in waxy corn were greater (p<0.05) than those in normal corn fed to growing pigs. After steam flaking, the ATTD of ADF in normal corn increased (p<0.05), while the ATTD of EE and NDF in normal corn decreased (p<0.05).

## DISCUSSION

The nutrient contents including DM, GE, EE, CP, crude ash, NDF, ADF, calcium, phosphorus, and starch were close between the two corn varieties, which were consistent with results from the previous study [1]. However, the structure and physicochemical properties of starch varied between waxy corn and the normal corn, with waxy corn having greater amylopectin content and degree of starch gelatinization. Starch gelatinization refers to the irreversible swelling of the starch granule during the digestion process [9]. The starch in corn accounts for 60% to 75% of the DM quality, which provides more than 60% of the total energy of corn [21]. Mohd-Azemi [22] used porcine pancreatic α-amylase in an *in vitro* study to measure the starch digestibility of different maize variety, and their results indicated that the digestibility of waxy corn starch was greater than that of normal corn. For growing pigs, Li et al [1] reported that the variety of corn had an impact on the nutritive values of corn and similar findings have been reported in other cereal grains [23–25]. In the current study, the waxy corn had greater DE and ME contents as well as the NDF and ADF digestibility. This result was consistent with Kotara’s reports [26], who found that with the increased gelatinization degree of corn starch, ATTD of dietary energy, NDF and starch had increased. One possible reason is that the processing technology improves starch digestibility of cereals [27]. Thus, waxy corn could also be used as a good energy source as a replacement of normal corn due to its greater amylopectin content and starch gelatinization degree.

In the feed industry, the nutritional values of feed ingredients usually improved after processing, such as dry, wet, cold, hot, puffing, or steam flaking. Generally, the starch gelatinization degree can reach 80% to 100% in the process of extrusion and puffing [2]. Previous research showed that the growth performance and nutrient digestibility of weaned piglets were improved with the increase of gelatinization degree of corn starch [26]. Therefore, purpose of granulation and puffing is to gelatinize the starch in the raw material to facilitate starch digestion and utilization. In this study, the steam procedure increased the contents of soluble NSP and the degree of starch gelatinization. Meanwhile, the DE and ME contents and the ADF digestibility of steam-flaked corn were greater than the normal corn, which agrees with results reported by Adeola and Bajjalieh [3], who demonstrated that puffing treatment had positive effects on nutrient digestibility of corn in pigs. A possible explanation is that steam flaking could release a certain amount of starch and protein from the starch-protein matrix and granule structure [10]. The effect of steam flaking on starch digestibility of corn in growing pigs is lacking in previous literature. In lactating cows, Plascencia and Zinn [28] found that steam flaking can raise the starch digestibility from 90% to 99% in corn. In feedlot cattle, Barajas and Zinn [29] also found that steam-flaked corn increased the total tract starch digestibility by 19% compared with dry corn. However, steam flaking decreased the EE and NDF digestibility. This might be related to the degree of corn steam compression as reported by Vicente et al [30], who found that heat processing of rice under mild conditions enhanced nutrient digestibility and productive performance of pigs, but severe processing of rice did not further improve nutrient digestibility or growth performance. Future studies are needed to explore the suitable degree for corn steam compression.

## CONCLUSION

This study revealed that both variety and processing procedure could influence the chemical composition, energy contents and nutrient digestibility of corn. Waxy corn could be used as a replacement of normal corn in diets for pigs because of its greater DE and ME values as well as NDF and ADF digestibility. Steam flaking of corn could improve the nutritional value when fed to growing pigs, but the appropriate degree of processing requires attention.

## Figures and Tables

**Table 1:** Ingredient and analyzed nutrient composition of experiment diets (%, as-fed basis)

Item	Waxy corn	Normal corn	Steam-flaked corn
Ingredient	-	-	-
Waxy corn	96.30	-	-
Normal corn	-	96.30	-
Steam-flaked corn	-	-	96.30
Dicalcium phosphate	1.70	1.70	1.70
Potassium carbonate	0.60	0.60	0.60
Salt	0.30	0.30	0.30
Antioxidants1)	0.10	0.10	0.10
Mineral and vitamin premix2)	1.00	1.00	1.00
Total	100.00	100.00	100.00
Analyzed nutrient			
Dry matter	87.70	88.62	90.83
Gross energy (kcal/kg)	3801	3785	3914
Neutral detergent fiber	18.14	16.51	11.02
Acid detergent fiber	3.57	2.79	3.14
Ether extract	2.95	2.77	2.35
Crude protein	7.58	7.23	7.48
Crude ash	3.76	3.57	3.49
Calcium	0.68	0.72	0.70
Phosphorus	0.44	0.43	0.44
Starch	69.11	70.17	73.23

1) Santoquin MAX (Novus International, Inc., St. Louis, USA), the compound antioxidant, containing no less than 10% ethoxy quinoline, no less than 3% butyl hydroxytoluene and citric acid.

2) Premix provided the following quantities per kilogram of complete diet: vitamin A as retinyl acetate, 5,512 IU; vitamin D_3_ as cholecalciferol, 2,200 IU; vitamin E as DL-alpha-tocopheryl acetate, 30 IU; vitamin K_3_ as menadione sodium bisulfite, 2.2 mg; vitamin B_12_, 27.6 μg; riboflavin, 4 mg; pantothenic acid as DL-calcium pantothenate, 14 mg; niacin, 30 mg; choline chloride, 400 mg; folacin, 0.7 mg; thiamin as thiamine mononitrate, 1.5 mg; pyridoxine as pyridoxine hydrochloride, 3 mg; biotin, 44 μg; Mn as manganese oxide, 40 mg; Fe as iron sulfate, 75 mg; Zinc as zinc oxide, 75 mg; Cu as copper sulfate, 100 mg; I as potassium iodide, 0.3 mg; Se as sodium selenite, 0.3 mg.

**Table 2:** Analyzed chemical composition of the three test ingredients (%, as-fed basis)

Item	Waxy corn	Normal corn	Steam-flaked corn
Bulk density (g/L)	726.47	726.49	367.90
Dry matter	87.25	88.02	90.32
Gross energy (kcal/kg)	3951	3,926	4,022
Ether extract	3.83	3.00	2.65
Crude protein	7.89	7.47	7.64
Crude ash	1.09	0.95	0.91
Neutral detergent fiber	20.84	19.04	18.53
Acid detergent fiber	3.45	3.14	3.36
Calcium	0.02	0.01	0.03
Phosphorus	0.16	0.16	0.16
Starch	70.33	73.12	76.32
Amylose	1.42	15.17	17.11

**Table 3:** The composition of dietary fiber, non-starch polysaccharides and starch gelatinization degree of test ingredients (%, as-fed basis)

Item	Waxy corn	Normal corn	Steam-flaked corn
Dietary fiber			
Insoluble	7.60	8.55	8.15
Soluble	0.40	1.10	0.90
Total quantity	8.00	9.65	9.05
Rhamnose			
Insoluble	0.024	0.022	0.020
Soluble	0.006	0.005	0.007
Total quantity	0.030	0.027	0.027
Fructose			
Insoluble	0.018	0.010	0.012
Soluble	0.002	0.001	0.003
Total quantity	0.019	0.011	0.014
Ribose			
Insoluble	0.051	0.034	0.024
Soluble	0.002	0.003	0.004
Total quantity	0.054	0.037	0.028
Arabinose			
Insoluble	1.473	1.417	1.483
Soluble	0.077	0.073	0.113
Total quantity	1.550	1.490	1.596
Xylose			
Insoluble	1.553	1.683	1.650
Soluble	0.061	0.057	0.243
Total quantity	1.614	1.740	1.893
Mannose			
Insoluble	0.234	0.190	0.240
Soluble	0.283	0.213	0.088
Total quantity	0.518	0.403	0.327
Galactose			
Insoluble	0.373	0.375	0.385
Soluble	0.034	0.031	0.278
Total quantity	0.407	0.407	0.663
Glucose			
Insoluble	2.062	1.956	2.082
Soluble	0.170	0.142	0.160
Total quantity	2.232	2.098	2.242
Non-starch polysaccharides			
Insoluble	5.79	5.69	5.89
Soluble	0.64	0.52	0.90
Uronic acid	1.07	1.05	1.02
Total quantity	7.49	7.26	7.81
Starch gelatinization degree	5.85	2.28	25.09

**Table 4:** The digestible (DE) and metabolizable energy (ME) contents and nutrients digestibility of test ingredients

Item	Waxy corn	Normal corn	Steam-flaked corn	SEM	p-value
DE (kcal/kg)					
As-fed basis	3,493^b^	3,424^c^	3,610^a^	0.09	<0.01
DM basis	4,004^a^	3,890^b^	3,997^a^	0.08	<0.01
ME (kcal/kg)					
As-fed basis	3,426^b^	3,353^b^	3,524^a^	0.09	<0.01
DM basis	3,926^a^	3,809^b^	3,901^a^	0.08	<0.01
Digestibility (%)					
Dry matter	89.47	88.20	89.72	0.33	0.14
Ether extract	59.35^a^	56.96^a^	41.97^b^	2.16	<0.01
Crude protein	76.56	74.19	80.00	1.06	0.07
Crude ash	60.20	54.24	51.70	1.56	0.06
Neutral detergent fiber	83.90^a^	66.47^b^	56.55^c^	2.93	<0.01
Acid detergent fiber	62.62^a^	45.09^b^	58.75^a^	2.49	<0.01
Calcium	61.73	59.58	56.96	1.89	0.61
Phosphorus	53.84	51.07	51.64	1.45	0.73
Starch	99.58	99.60	99.82	0.05	0.08

SEM, standard error of the mean; DM, dry matter.

a–c Within a row, means followed by the same letters are not different at p<0.05.
